# Ripple Effects Mapping: Evaluating Multilevel Perspectives and Impacts of a Statewide Community–Academic Partnership Network on Covid‐19 Health Disparities

**DOI:** 10.1111/hex.70446

**Published:** 2025-09-29

**Authors:** Evelyn Vázquez, Isabel Duong, Borsika A. Rabin, Nicole A. Stadnick, Paul L. Watson, Kelli L. Cain, Maria Pozar, Ann Cheney

**Affiliations:** ^1^ Social Medicine, Population and Public Health University of California Riverside Riverside California USA; ^2^ College of Natural and Agricultural Sciences University of California Riverside Riverside California USA; ^3^ Herbert Wertheim School of Public Health and Human Longevity Science University of California San Diego La Jolla California USA; ^4^ UC San Diego Altman Clinical and Translational Research Institute Dissemination and Implementation Science Center University of California San Diego La Jolla California USA; ^5^ Department of Psychiatry University of California San Diego La Jolla California USA; ^6^ Child and Adolescent Services Research Center San Diego California USA; ^7^ The Global Action Research Center San Diego California USA; ^8^ Conchita Servicios de la Comunidad Mecca California USA

**Keywords:** California, community‐engaged research, Covid‐19, health disparity populations, ripple effects mapping

## Abstract

**Introduction:**

Community–academic partnerships played an important role in addressing Covid‐19‐related health disparities in historically marginalised groups such as racial ethnic minorities and low‐income and rural communities in the pandemic. Part of the federal response involved establishing regional community academic networks that engaged highly impacted communities in health disparities research. The statewide Share, Trust, Organize, Partner COVID‐19 California Alliance (STOP COVID‐19 CA) network was part of the federal response.

**Methods:**

In spring 2024, ripple effects mapping (REM), a participatory action research method, was used to conduct an evaluation of the impact of the STOP COVID‐19 network on the capacity of community–academic partnerships to carry out Covid‐19‐related health disparities research. This method uses group interviews to capture direct and indirect outcomes, that is, ripples, of community‐based programmes. Short‐, medium‐ and long‐term changes and conditions related to community–academic partnerships in the statewide network were mapped onto the spheres of influence of the social ecological model.

**Results:**

A total of 24 participants took part in one of three REM sessions. Community and academic partners were represented in all sessions, and most had been involved in community‐engaged research for 3 to 10+ years. Most identified as female, Hispanic/Latino, and between the ages of 40 and 49. Qualitative analysis of sessions indicated that most changes occurred at the individual and interpersonal levels and involved medium‐term changes (e.g., increased capacity to partner in research and shared understanding). Neighbourhood‐ or community‐level changes included identification of culturally and linguistically responsive intervention and dissemination efforts (e.g., promotora model). Policy and built environment conditions reveal the inequities in higher education and the need for structural‐level changes to university infrastructure and grant administration.

**Conclusion:**

Most outcomes were observed at the individual and interpersonal (group) levels and involved primarily medium‐term changes. However, the network itself served as a platform to discuss the need for structural‐level changes within university infrastructure to facilitate community‐engaged scholarship. Such networks have the potential to facilitate capacity building for community–academic partnerships to collaborate in health disparities research that can generate evidence to move forward public health policy change.

**Patient or Public Contribution:**

Community partners, including grassroots leaders and staff of community‐based organisations, were involved in the development of the research questions, the design of the study, and data collection, analysis and interpretation of the findings. Community partners also contributed to manuscript development.

## 1 Introduction

The Covid‐19 pandemic exposed significant disparities in health outcomes, particularly among historically marginalised communities. Racial‐ethnic minorities and low‐income communities, including Latinx (especially undocumented individuals), Black/African American and Native American communities, experienced disproportionately high rates of Covid‐19 infection, hospitalisation and mortality [1]. Research underscores that these health disparities were deeply rooted in structural inequalities [2].

Research highlights several factors contributing to exacerbated health disparities during the Covid‐19 pandemic. Housing conditions, neighbourhood density and geographic location were significant contributors to the spread of Covid‐19 [3]. Factors such as systemic racism, xenophobia and economic instability (precarious working conditions and income inequality, etc.) further heightened vulnerability among these communities [4, 5]. Community–academic partnerships were instrumental in identifying these issues and implementing culturally responsive interventions. These collaborations informed culturally responsive interventions by prioritising language and terminology that resonate with the community rather than solely aligning with academic or public health discourse [6, 7]. These teams served as trusted sources of information and developed public health messaging and programming that aligned with shared values within communities and built on community assets to address healthcare needs in historically marginalised communities during the pandemic [8, 9].

Community–academic partnerships that used principles of community‐based participatory research (CBPR) approaches played a pivotal role in addressing these disparities by integrating community priorities as well as knowledge and cultural sources of strength with academic rigour [9, 10]. CBPR involves shared leadership and decision‐making along with equitable allocation of resources [11]. This collaborative approach draws on the expertise of community and academic partners to identify solutions and address healthcare needs meaningful to the community. In the Covid‐19 pandemic, regions with a strong presence of community–academic partnerships saw an increase in vaccinated adults, suggesting that these partnerships facilitated more effective public health responses and tailored interventions that acknowledged and addressed the unique barriers and challenges faced by marginalised communities [12].

CBPR and community–academic partnerships were crucial in designing and executing effective interventions to mitigate health disparities. Community–academic partnerships facilitated the collection of more accurate and relevant data and ensured that interventions were culturally and contextually appropriate [13], by engaging directly with affected communities, researchers and practitioners tailored health communications [14], increased vaccine uptake [15, 16], and identified specific barriers to Covid‐19 testing and vaccination [17, 18] among historically marginalised communities during the pandemic.

The Covid‐19 pandemic underscored the importance of community–academic partnerships in mitigating the spread of Covid‐19 and reducing the effects of the pandemic on historically marginalised communities. While research has assessed their contribution to Covid‐19 awareness and public health responses as well as impact on Covid‐19 vaccine uptake [7, 12, 15], a better understanding of the impact of community–academic partnerships on collective capacity to address health disparities in historically marginalised communities is needed [19]. This manuscript addresses this gap by reporting on the evaluation of the impacts of a statewide network on building the capacity of community–academic partnerships to collaboratively address health disparities in marginalised communities during the Covid‐19 pandemic.

## 2 Materials and Methods

The research was part of a larger mixed‐methods evaluation that included ethnographic observations of community–academic partnership dynamics, online surveys on partnership engagement practices, and qualitative group interviews that were carried out by members of two community–academic partnerships in the statewide network Share, Trust, Organize, Partner COVID‐19 California Alliance (STOP COVID‐19 CA). This statewide alliance, STOP COVID‐19 CA, was established in 2020 with funding from the National Institute of Health (NIH) Community Engagement ALliance (CEAL) initiative, which was part of the federal government's response to mitigating the effects of the pandemic on minoritized communities [9].

One of the final phases of Covid‐19 work carried out by the STOP COVID‐19 CA network involved the development and convening of a learning community to share best practices, disseminate study findings from across the network, and contribute to the development of potential public health policy change. We used ethnographic observations and surveys on partnership engagement practices to document engagement in the learning community. We found that community and academic partners in the meetings perceived themselves to contribute about the same amount of time, and both rated their engagement in network activity favourably. As a next step, we used ripple effects mapping (REM) [20], a participatory action research method that assesses the impacts (changes and consequences) of a programme, to examine the network's impact on the capacity of community–academic partnerships to carry out health disparities research. This paper reports on these findings.

Community and academic partners with expertise in ethnographic and qualitative research carried out data collection and analysis. This included a community investigator with over 7 years of experience facilitating group interviews and two academic investigators with expertise in qualitative research who designed and conducted the evaluation with support from student research assistants. All team members spoke Spanish and contributed to data collection and analysis.

The objective of the research was twofold: (1) use REM to identify the impact (changes and consequences) of a statewide community‐engaged alliance on community–academic partnerships carrying out Covid‐19 research with health disparity populations and (2) visually represent identified impacts over time and contextualise them across social‐ecological levels. Before the start of research, we obtained ethical approval from the University of California (UC) Riverside Institutional Review Board (IRB). The IRB approved the study as ‘minimal risk’ and expedited. All data collection was conducted virtually; as such, a waiver of signed consent was obtained so that participants could provide electronic consent via an online consent form.

### 2.1 Setting

This study was carried out in California and assessed the impact of the STOP COVID‐19 CA network on health disparities research during the pandemic. The primary goal of STOP COVID‐19 CA was to address gaps in Covid‐19 information, vaccine trial participation and vaccination accessibility within underserved communities across California [9]. A community engagement framework that emphasised culture and context, community‐informed recruitment and retention strategies, and capacity building guided the alliance's activities (see Casillas et al. 2022 for more information on this framework) [9]. This statewide network included 11 academic medical centers and over 75 community collaborators, including community advocates, health system, non‐profit leaders, faith‐based organisations, grass roots organisations, government officials from state and county public health departments, community‐based media, and immigrant advocacy groups from 14 counties covering almost 75% of the population in California. The alliance created a research network in California that addressed the effects of the pandemic in some of the hardest hit communities in California, including rural Latinx and Indigenous Mexican farm workers, immigrant and refugee communities, Black/African Americans, Native Hawaiian and Pacific Islanders (NH/PIs), Native Americans, and Asian Americans.

The 11 sites included: San Diego State University, Scripps, Stanford, UC Davis, UC Irvine, UCLA, UC Merced, UC Riverside, UC San Diego, UC San Francisco and the University of Southern California. Within the first 2 years of the pandemic (August 2020 to December 2021), the STOP COVID‐19 CA network administered surveys to 11,000 people in California, conducted 133 focus groups, partnered in 29 clinical trials, and held over 300 community events (e.g., town halls, Covid‐19 testing and vaccine events) [9]. Other key aspects of the network include engagement of community health workers (CHWs)/promotoras in research and public health education and promotion. CHWs/promotoras served as members of community–academic partnerships bringing their expertise in community health needs and recruitment strategies, capacity to assist with data collection and analysis (e.g., focus group facilitators and administration of surveys), and knowledge of best practices to disseminate study findings and public health information to marginalised communities [21].

### 2.2 Participant Eligibility and Recruitment

This study focused on the experiences and insights of members of the 11 community–academic partnerships in the STOP COVID‐19 CA network. Participants were eligible if they were: (1) 18 years of age or older, (2) spoke English or Spanish and (3) identified as a member of a community–academic partnership in the STOP COVID‐19 CA statewide network. Convenience sampling, a non‐random and non‐probability sampling method based on convenience and access to alliance members, was used to recruit participants [22]. Our approach involved two recruitment strategies. First, we recruited participants via a learning community formed as part of an alliance activity that focused on sharing best practices, disseminating study findings and identifying potential next steps for public health policy change recommendations. This learning community marked the final phase of the alliance's Covid‐19 research focus. We anticipated that at least 200 members, including community and academic partners, across the 11 sites would participate in the learning community. However, fewer than 50 alliance members participated, limiting the recruitment pool. We invited all 50 members to join the study; however, only about a quarter of the members joined the study. We recruited additional participants by sharing the study flyer with alliance members during statewide meetings and via emails.

### 2.3 Data Collection: REM

We collected data in March 2024 using Zoom video conferencing. We chose this platform because of its familiarity among members of the network, which was used throughout the pandemic to bring together community academic partners in the statewide network. Additionally, the Zoom breakroom features permitted placement of participants into separate rooms for small group discussions.

We selected REM to evaluate the activity of community–academic partnerships in the statewide network due to its participatory nature and because it captures both the direct and indirect outcomes—or ‘ripples’—of community‐based programmes. The method encourages reflection among participants, revealing intended and unintended changes resulting from their involvement in a community–academic partnership in the statewide network. REM also visualises the cascading effects of community‐engaged initiatives. Per REM field guides, there are four core elements in REM: (1) Appreciative Inquiry, which focuses on identifying and building on strengths and successes; (2) a Participatory Approach, ensuring active involvement from staff and participants in programmes/projects; (3) Interactive Group Interviewing and Reflection, which facilitates dynamic discussions and collective insights; and (4) Radiant Thinking, or mind mapping, which visually organises and connects ideas to illustrate the broader impact of interventions [20, 23]. Three approaches to REM have been developed, including web mapping, in‐depth rippling, and theming and rippling. These approaches offer distinct ways to assess the community impact of interventions or programmes that involve diverse participants and collaborators. Web mapping organises outcomes into short‐, medium‐ and long‐term impacts, to visually map changes that emerged as part of the partnerships. In‐depth rippling focuses on identifying the most impactful chains of events, ensuring that the deepest and most transformative effects of an intervention are captured. Theming and rippling collects a broad range of participant‐reported impacts, categorises them into overarching themes and then examines the ripple effects within those themes. The three approaches allow evaluators and stakeholders to collaboratively explore how programmes generate both intended and unintended effects and outcomes, creating a comprehensive picture of community transformation. We used the in‐depth rippling approach of REM as it allows for the identification of short‐, mid‐level and long‐term outcomes [24, 25].

We conducted three REM sessions in March 2024, with participants from 8 of the 11 community academic sites involved in the STOP COVID‐19 CA network. These sessions, conducted in both English (*n* = 1) and Spanish (*n* = 2), were facilitated by trained facilitators and involved peer‐to‐peer interviews followed by group discussions.

#### 2.3.1 Peer Interviews

The first step involved conducting peer appreciative inquiry interviews in which two peers interviewed each other. Peers were preassigned to breakout rooms before the start of each session, and community and academics were purposively paired up when possible. Each peer served as an interviewer, asking their peer the following two prompts: (1) What is your role within the community–academic partnership in the statewide STOP COVID‐19 CA network? and (2) How have you used the information learned or skills developed as part of this network? In the role of interviewer, participants were instructed to take notes and jot down key points to share with the full group. After completing the first round, the roles were reversed, with the interviewer becoming the interviewee and vice versa, allowing for a mutual exchange of insights. In total, each pair had 10 minutes (5 min per participant) to complete the peer interviews.

#### 2.3.2 Reporting on Group Discussion

Participants rejoined the main Zoom session, and each pair was given 5 min to present their story. During the group discussion, facilitators guided participants through a structured process to share insights from their peer interviews. Using an interview guide, facilitators prompted participants to discuss their experiences within the network, focusing on strengths (e.g., knowledge and skills gained) and weaknesses (e.g., challenges encountered). As each pair reported their findings, the facilitator asked follow‐up questions to elicit deeper reflections and additional details related to the broader impacts of the network on community‐engaged research. As stories were shared, the facilitator asked: (1) How has this changed your team and its capacity? (2) What is everyone doing differently now? and (3) How have relationships between the community and the university evolved? These discussions focused on how the network influenced team dynamics, capacity building and the strengthening of relationships between community partners and academic institutions. Depending on the number of pairs, the discussion lasted between 45 and 75 min, and key insights were visually captured using Miro, a virtual mapping tool. As participants shared their insights, facilitators captured those insights on a Miro board to create a visual map of participants' responses. This map served as a focal point for further discussion and analysis, allowing participants to see how their individual and collective actions contributed to broader changes.

#### 2.3.3 Reflection

Participants then reflected on the generated map and discussed how their involvement in the STOP COVID‐19 CA network contributed to changes at multiple levels—individual, interpersonal, community and policy. The discussion lasted 10–15 min and was centred on several key questions: (1) What are the most significant changes represented on the map? (2) What stands out as the most interesting feature? (3) How can the map illustrate the capacity of teams in the STOP COVID‐19 CA network? (4) And how can the map help address health inequities for vulnerable communities?

#### 2.3.4 Socio‐Demographic Survey

Before the start of the REM sessions, participants completed a brief socio‐demographic survey via Qualtrics. The survey collected information about gender, age, birthplace, preferred language, ancestry/heritage, education level and community–academic partnership role.

### 2.4 Data Analysis

The data collected during the REM sessions included transcripts of the peer and group interviews. The analysis was guided by the social ecological model (SEM). Originally developed by Bronfenbrenner [26], SEM highlights the dynamic interplay between individual, interpersonal, organisational, community and policy‐level factors, emphasising that health behaviours are not determined solely by individual characteristics but are shaped by broader social and environmental contexts [27, 28]. In public health, this model guides the design of interventions that target various levels of influence to maximise impact, as seen in efforts to improve agricultural safety [29] and healthy eating [30]. This approach allowed for a comprehensive understanding of the project's impact, highlighting short‐term, medium‐term and long‐term changes. Our categorisation of the three changes follows the framework proposed by Chazdon et al. [20], which is specific to the Web Mapping variation of the ripple effect mapping approach. According to this conceptualisation, short‐term changes refer to shifts in knowledge, skills or attitudes; medium‐term changes refer to behavioural changes (it can be at the individual, group or institutional level); and long‐term changes reflect broader systemic impacts, such as changes in the infrastructure or policy level.

The first author, a qualitative expert and one of the facilitators, read the transcripts line‐by‐line and conducted content analysis guided by the SEM, with primary codes corresponding to four levels: individual, interpersonal/group, community/neighbourhood and policy/built environment. Each level was further categorised according to the three types of changes defined in the Web Mapping variation of the REM approach: short‐term changes (e.g., knowledge, skill or attitude changes), medium‐term changes (e.g., behavioural modifications) and long‐term changes (e.g., structural or policy‐level impacts). To ensure consistency and rigour in our qualitative analysis, we developed an initial codebook inductively based on themes that emerged from the REM session data, while maintaining alignment with the SEM framework. We applied the initial coding framework to a subset of the data and refined the code definitions through an iterative process.

**Figure 1:**
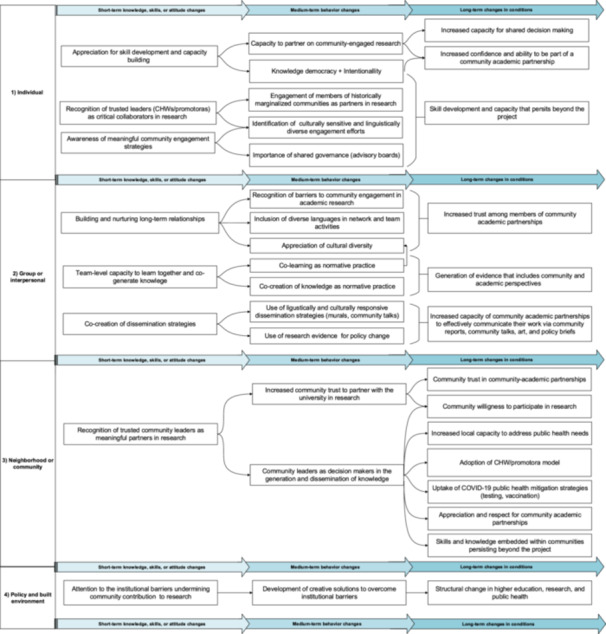
Ripples associated with the STOP COVID‐19 CA network by short‐, medium‐ and long‐term changes.

Following this analysis, the first author created a map per session, divided into four sections to capture the ‘ripples’ associated with the STOP COVID‐19 CA network (see Figure 1). The first section of the model outlines the four levels of SEM. The three following sections reflect in‐depth ripples aligned with the REM approach. The second section details short‐term changes in knowledge, skills or attitudes. The third section captures medium‐term behavioural changes. Finally, the fourth section addresses long‐term changes in conditions. This structured REM approach enhanced the trustworthiness of the data collected by providing a clear and comprehensive framework for conducting a cross‐case analysis and illustrates the impacts of community–academic partnerships involved in the statewide network [31].

As a last step, we conducted a frequency analysis by quantifying and categorising the ripples (i.e., impacts and outcomes) of the network on the capacity of community–academic partnerships in the network. This analysis focused primarily on the quantification and distribution of reported outcomes across the levels of the SEM. It has been effectively used in previous studies to highlight outcome distributions across different levels of the SEM [25]. Visualising the data using this approach permits a systematic way to identify the impacts of the network on outcomes.

## 3 Results

### 3.1 Participant Characteristics

Of the 24 REM session participants, all completed the socio‐demographic survey (see Table 1). Most participants identified as female (79%). Participants were between the ages of 20 and 59 years. A total of 63% of participants self‐identified as Hispanic/Latino and 29% White; 63% preferred English and 42% Spanish as their primary language. The highest level of education among participants ranged from elementary school to graduate school/advanced degree. There was a nearly even distribution between community (58%) and academic (42%) partners, with the majority having participated in a community–academic partnership for several years (38% involved for 3–5 years and 29% involved for 10+ years) and 33% indicated they were new to this type of partnership and research.

**Table 1:** Characteristics of participants in REM sessions.

Characteristics	Total *N* = 24	%
Gender		
Male/Man	3	13
Female/Woman	19	79
Trans male/Trans man	1	4
Different identity	1	4
Age		
20–29	5	21
30–39	4	17
40–49	8	33
50–59	7	29
Heritage/Ancestry		
Indigenous Latin American	2	8
Asian/American Asian	1	4
Black	1	4
White	7	29
Hispanic/Latino	15	63
Birthplace		
The United States	12	50
Latin American (Mexico and Central America, South America)	11	46
Europe	1	4
Preferred language		
English	15	63
Spanish	9	37
Highest education level		
Never attended school	0	—
Only attended kindergarten	0	—
Attended elementary or primary school	1	4
Attended middle or secondary school	1	4
Completed high school or obtained a GED (or high school graduate)	2	8
Completed some community college or technical school	3	12
Completed a 2‐year associate's degree	0	
Completed college or 4 years of university	8	33
Completed graduate school (or advanced degree)	9	38
Role in community–academic partnership		
Community partner	14	58
Academic partner	10	42
Community Partnership Position (out of 14)		
Community investigator	2	8
Community health worker	6	25
Project coordinator	2	8
Research assistant	0	
Administrative staff	1	4
Other	3	13
Academic Partnership Position (out of 10)		
Principal investigator or co‐investigator	2	8
Student–research assistant or researcher	3	13
Project coordinator	2	8
Research assistant (staff, not student status)	2	8
Administrative staff	1	4
Longevity of involvement in community‐engaged research		
1 year or less	4	17
2 years	2	8
3–5 years	9	38
5–10 years	2	8
10 or more years	7	29

### 3.2 Qualitative Findings: Changes Across Multiple Levels of Influence

We mapped the changes and impact across the multiple levels of influence according to the SEM framework (see Figure 1). At the individual level, participants reported an increased capacity to engage in research addressing public health needs in historically marginalised communities. Notably, for about a third of the community collaborators, being an integral member of a community–academic partnership in the STOP COVID‐19 CA network was their first engagement with public health efforts. At the group/interpersonal level, there was an increase in the capacity of the community–academic partnership to address local public health needs, long‐term relationship building and co‐creation of dissemination strategies. At the community level, there was a notable increase in trust towards research, public health initiatives and trusted community leaders such as CHWs/promotoras as liaisons between the community and the community–academic partnership. At the structural/policy level, there was an emphasis on changing the university infrastructure to be more flexible and considerate of community partners and their critical role in community‐engaged research with health disparity populations. There was also an emphasis on using findings from the network to inform public health policy change.

**Figure 2:**
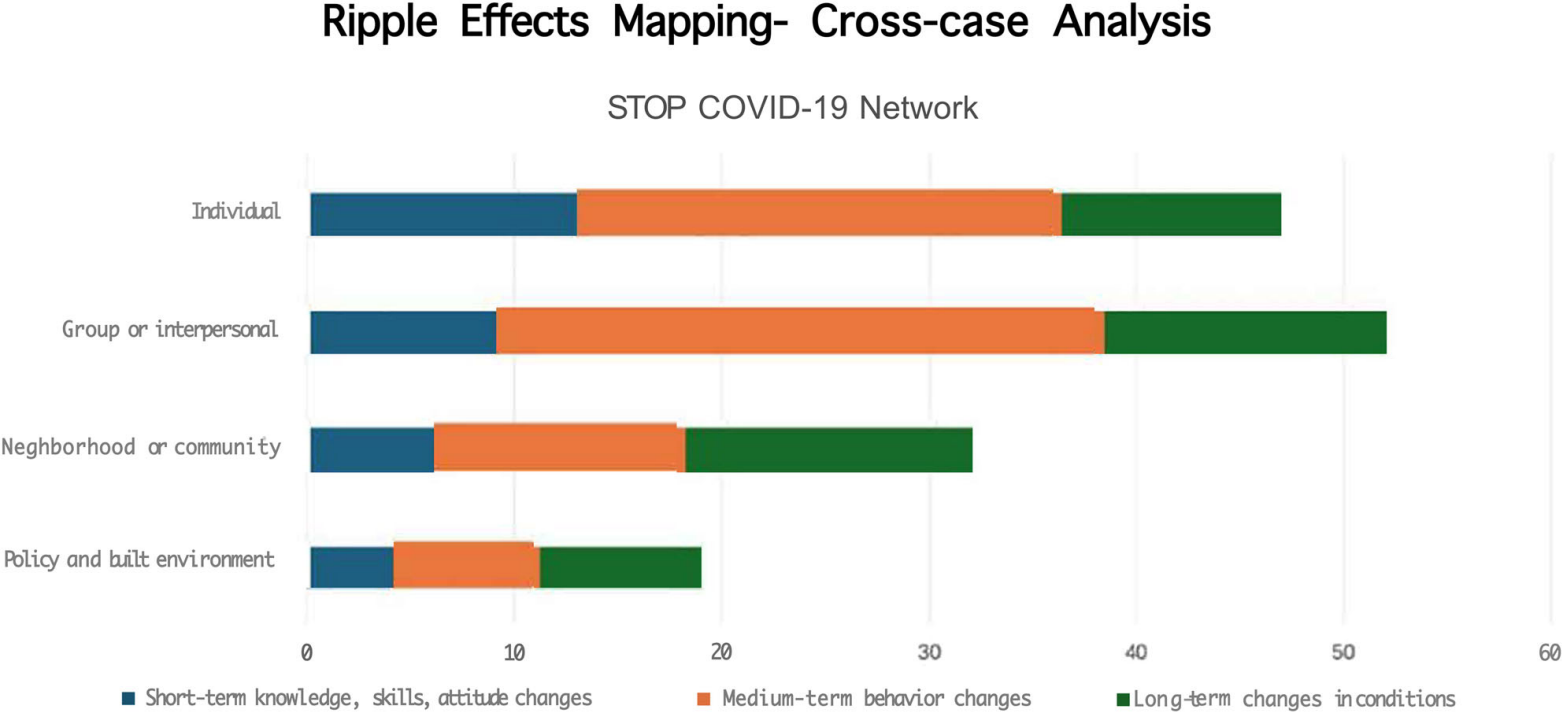
REM—A cross‐case analysis.

Results from the frequency analysis further substantiate the perceived changes and impact across participants' narratives. From Figure 2, we learned that most outcomes from the partnerships of the statewide network were observed at the individual and interpersonal (group) levels. These outcomes primarily included medium‐term changes, such as modifications in partner behaviours and group dynamics. This suggests that the study identified how the participation of community–academic partnerships in the statewide network facilitated behavioural shifts among individuals and within small community groups. While participation in the network may have also facilitated structural‐ or policy‐level transformations in public health and institutions of higher education, our use of REM did not permit an in‐depth, exhaustive understanding of the role of the network in producing such results.

#### 3.2.1 Individual‐Level Changes

At the individual level, involvement in the STOP COVID‐19 CA network led to meaningful behaviour changes. Some of the changes include short‐term knowledge, skills or attitude changes, including the recognition of trusted community leaders such as CHWs/promotoras as collaborators in research, and most changes occurred at the medium term, such as an increased capacity to partner on CBPR or community‐engaged research, while some long‐term changes in conditions, including the importance of shared decision‐making, occurred.

Community leaders gained confidence in their own abilities to partner in research and project activity regarding Covid‐19. A CHW/promotora reflected on how her involvement in the network work fostered changes for herself, at the individual level:

The truth is, I do not know how to study well [formal education], but [this project] helped me a lot because I was very shy…. The community taught me to lose the fear of speaking [in public], and what I learned … was that the university always helped me during meetings, giving me more confidence to share the information.

As this participant shared, the community–academic partnership in which she was involved fostered capacity building and empowerment by providing collaborators, such as CHWs/promotoras, with the skills and confidence they needed to meaningfully partner in research.

In addition to short‐term knowledge, skills or attitude changes, participants also reflected on medium‐term behaviour changes at the individual level involving the valuation of shared governance, such as through community and stakeholder advisory boards. An academic partner shared, ‘I would say this [project] really taught me about CPBR … when you are doing things, you really need to be focusing on the community that you are also trying to make change in’. Participants also talked about the importance of community advisory boards. A community partner said:

Participating in this STOP COVID‐19 CA project gave me confidence in understanding firsthand what a community advisory board is, what the purpose of it is, which I take it as having a structured way so that you have a collaboration that's representative of the community. I guess it just gave me more confidence that something like this could be done.

Finally, participants expanded on how the project facilitated long‐term changes in conditions, such as the engagement of members of historically marginalised communities in research addressing the needs of their community and the potential for knowledge generated to persist beyond the project. A community partner commented:

Learning how to speak to different people and different backgrounds and cultures. Seeing them as people and making sure they have the resources that they need to take care of themselves and their communities. To carry out that knowledge that they gained to future generations as well.

#### 3.2.2 Group or Interpersonal Level Change

Changes at the individual level were closely intertwined with changes at the group level. As individuals gained new knowledge, skills or perspectives, these personal changes influenced their interactions within the group and across the network, fostering more collaboration, trust and shared understanding, which led to building and nurturing long‐term relationships, team‐level capacity to learn and generate knowledge together, and the co‐creation of dissemination strategies. In turn, these enhanced group dynamics reinforced individual growth, creating a feedback loop where personal and collective progress mutually supported one another. For instance, a community partner shared how involvement in the network fostered individual‐ and team‐level growth:

[The project] has been a big change in learning, knowledge, community work, and everything we have been able to share and bring to our communities. I think we have changed a lot and have grown both as a team and as individuals.

Some of the changes include short‐term knowledge, skills or attitude changes, including the need to nurture long‐term relationships. An academic partner reflected on the need to compensate people for their time to nurture long‐term relationships: ‘We mentioned everything from how to get people paid and making sure people are paid on time, making sure that the community felt they had a space at the table’. Medium‐term behaviour changes include the need to identify effective culturally responsive and linguistically diverse dissemination efforts. These included community talks, murals and policy briefs that were tailored to meet the needs of diverse populations. An academic partner said,

Working with artists, in this case, muralists to find creative ways to share the work, share the findings. I think, what really struck me was, she [participant] mentioned the mural as a means for community healing, which I thought was really beautiful.

Another academic partner emphasised the importance of sharing findings in ways that resonate with the community:

[M]aking sure that the community is at the forefront of what we are doing and that the research does not get lost in the academic bubble … a peer‐reviewed paper … is not necessarily the best way for the information to be used by the community.

Many discussed the importance of co‐creating culturally responsive and linguistically diverse dissemination strategies to address health disparities, indicating they ensure research findings are accessible and relevant to the communities most affected.

Participants also reflected on long‐term changes at the group or interpersonal level, such as the importance of shared decision‐making, trust among community academic team members, and increased capacity to translate community research findings in ways meaningful to the community. A community partner mentioned:

We are a very community‐based organization, but this program helped us to develop … our academic side, as you say. It helped us to really search for and find the resources that we needed to get the information across to our clients who come from [marginalised] backgrounds, whether they had lack of education or they're immigrants or refugees and they didn't have access to any of that. This really helped build our resource capacity and make sure that they have the resources and the knowledge that they need to make an informed decision for their health and their community.

Others talked about how the network influenced the work of community–academic partnerships by pushing them to use research findings to initiate public health policy change. An academic partner commented:

[The community–academic partnership] were able, through what they learned, to help develop policy and practice briefs … about best practices and ways to help increase uptake in communities. They learned about what barriers existed and what the needs of community members were … ultimately helping improve access and improve health.

This quote illustrates how short‐term changes in attitudes around dissemination strategies led to the long‐term, group‐level changes regarding the importance of policy briefs on health inequity in historically marginalised communities to disseminate information on best practices for the uptake of Covid‐19 mitigation strategies to reduce Covid‐19‐related health inequities in historically marginalised communities.

#### 3.2.3 Community/Neighbourhood Level Changes

On the community/neighbourhood level, the REM sessions revealed an enhanced capacity for community‐based and health equity research and trust in research through increased awareness and recognition of the benefits of community‐engaged research for health equity efforts. Participants emphasised the importance of recognising and respecting the strengths of the communities involved and addressing the unique health needs of historically marginalised populations. Changes included short‐term shifts in knowledge, skills and attitudes, particularly regarding trusted community leaders. The following quote from a community partner illustrates this point: ‘You could not do education or research or anything without trusted messengers and trusted advocates’.

Participants also reflected on the importance of partnering with leaders of historically marginalised communities, so community members see themselves reflected in research and public health efforts. An academic partner said:

I would say for the Black community, it was receiving the message from someone who looks just like them. During this project, we noticed, based on responses, that the Black community really listens when the message is coming from someone who looks like them.

Similarly, this academic partner made the following remark regarding trusted community leaders:

[The community–academic partnership] engaged those communities to learn what barriers existed, what opportunities existed for delivering accurate information to those communities. They identified who trusted messengers are in those communities and the opportunity represented by partnering with those folks to make sure that folks had accurate information from people they trust and in the languages that they needed information in.

In addition to trusted leaders, participants also talked about the value of the CHW/promotora model in which CHWs/promotoras built trust with members of low‐income, non‐English speaking communities of colour. The active participation of community leaders and CHWs/promotoras in decision‐making processes was identified as a key factor in fostering trust and facilitating the exchange of knowledge within historically marginalised communities. These efforts were instrumental in increasing Covid‐19 testing and vaccination rates in the targeted communities. A community partner said:

The promotoras model … I think that is a big change, because before the pandemic, at least I had never heard of work that included promotoras. It is the most effective way to reach vulnerable and rural communities…. I have learned that these communities have a lot of trust in their promotoras. Using promotoras is the most effective way to share information, encourage vaccination, and communicate medical knowledge from academic people … because without them, the information and announcements being made would not reach these communities.

This quote illustrates a medium‐term change that stemmed from CHWs/promotoras, who are seen as trusted members of their community and known to disseminate trustworthy information. This community partner commented, CHWs/promotoras helped to develop trust in public health information:

Our promotoras, are somebody [community members] know … a neighbor, a mom, somebody in the community … they are able to trust that person with information they are sharing. Going door‐to‐door really helped, create that trust and continue to be consistent when sharing information.

The CHW/promotor model significantly expanded community–academic partnerships' reach while building community trust and fostering stronger partnerships in community‐engaged research, which illustrate long‐term shifts in carrying out community‐engaged research with health disparity populations.

Other long‐term shifts include practices around the dissemination of study findings so that evidence generated via research can be accessible and used by the community, which contributed to the community holding more trust in community–academic partnerships. A community partner commented:

[By] providing information to people in my community and showing that it was not just my words, but that the university and [a community–academic partnership] were involved, the community began to trust [the information I was sharing]. The information we shared with them was seen as accurate, and they realized it was helping them.

Similarly, participants highlighted how the work of the community–academic partnership dispelled myths and built community trust in academic research and public health. One academic partner shared:

I would say the community is more open to engaging folks at the university. We all know of the Tuskegee experiment and other things that had gone wrong with the government … we went out there and dispelled a lot of myths and were able to gain the trust of the community. With doing so, we still have those relationships with those community members. We are able to continue working with them. That was a huge plus for us, a great start to working with the community and gaining our trust.

Changes within the neighbourhood/community sphere often focused on growth in collective knowledge and collaboration, which helped improve local capacity, community trust in research and public health, and encouraged the adoption of Covid‐19 mitigation behaviours (e.g., testing and vaccination) within communities. This also contributed to long‐term changes centred on community‐engaged research as a method to build local capacity to address public health needs.

Participants also described how the project helped develop policy and practice briefs to influence local public health initiatives. Another academic partner commented:

[T]hey worked on projects or programs to help vaccinate folks in the [name of region]. They were able, through what they learned, to help develop policy and practice briefs that they shared with the health departments and other leaders about best practices and ways to help increase [vaccine] uptake in communities. They learned about what barriers existed and what the needs of community members were so that they could then share that information back and ultimately help improve access and improve health.

#### 3.2.4 Policy and Built Environment Changes

Participants reflected on the potential of the national NIH CEAL initiative to drive structural changes in higher education infrastructure that would better recognise the value of community in research with health disparity populations. By prioritising community–academic partnerships, the project reduced institutional barriers and promoted health equity through community‐engaged research. A community partner highlighted the importance of flattening hierarchy to overcome these barriers:

Within our own organizations and especially at the university where hierarchy is much more pronounced … if a learner [community partner] had an idea, it was respected … as powerfully as if it were faculty or the [principal investigator]. If a learner [community partner] or a staff member said, ‘I want to lead this particular publication’, they did. A lot of flattening of the hierarchy and respect for one another, regardless of where we sit in our own hierarchies.

This reflection highlights how engagement in the network ‘flattened’ hierarchy by valuing the expertise of community and academics in addressing the health disparities in the pandemic and fostering a collaborative atmosphere to encourage more effective public health interventions through equitable partnerships. Participants also talked about changes in the university infrastructure and administration to facilitate both grant writing and the contracting of community partners, an academic partner said:

Learning how to deal with logistical challenges, grant writing, etcetera, and working together with communities on that. It has changed our work in that we are constantly looking for better ways of working with the university infrastructure and administration to facilitate the community partners' contracting, both at the front and end. When we are writing proposals, we encourage them to provide more guidance. We work with the fund managers to provide more explicit guidance to our community partner on how to put together the paperwork that they need for subcontracts. Then also on the back end, when we are in the stage of payments and billing. While I do not think that it is ideal, but we are definitely feeling more empowered by working in this group to push back and look for more creative solutions for these contracting and proposal‐writing challenges.

According to participants, the STOP COVID‐19 CA network and being part of the NIH CEAL initiative offered a unique opportunity to identify ways to foster structural change in higher education. By creating spaces where hierarchies are intentionally flattened, the network encouraged collaborative learning and decision‐making across different roles.

### 3.3 Increasing the Impact of Community–Academic Partnerships

In the process of sharing the significant changes and impacts of the network on community–academic partnerships, participants talked about what needs to improve for partnerships and the network to be more impactful. This included greater recognition of community collaborators (e.g., CHWs/promotoras and CBOs), more funding, increased integration of findings from community‐engaged research into public health policy and practice, and changes within higher education to support community‐engaged research. A community partner commented:

I think they [university] recognize the work of the promotora [CHW], but they did not fully acknowledge us. We were the first to step up for the vaccine. When we heard the myths and fears from the community, about being implanted with a chip or that [the government] wanted to eliminate us, we were out there. I still feel that the work promotoras did—not just in our organization, but in all organizations—has not been fully valued.

Participants discussed the need for greater recognition of the contributions made by CHWs/promotores as well as CBOs in historically marginalised communities in challenging misinformation and building trust in research and public health. They expressed the need for more visible appreciation (e.g., university badges and names on university websites) and institutional support (e.g., more efficient payment structures) for collaborators to acknowledge their fundamental role in the success of engaging historically marginalised communities in public health research.

In addition, participants reflected on the importance of increased budgets for community‐based organisations to facilitate long‐term involvement and sustain community–academic partnerships. For instance, a community partner commented:

As nonprofit community‐based organizations, we are asked to participate in a lot of things, and we do participate in a lot of things. We do a lot of collaborations, we do a lot of networking, we do a lot of coalitions. A lot of it we do because we feel that that is our role. Our role as a connector and our role is to meet somebody and say, ‘Oh, you are doing X, Y, Z, you should talk to this person,’ or, ‘Let me introduce you to so and so and try to generate synergies’…. I love that about our nonprofit and our mission and the work we do, but that does not pay the bills, and so having stipends for the community‐based organizations to allow them to spend the time to interact with the network I think is really important. Like I said, I would love to be super active and be involved in all these meetings and see what is going on. I cannot because I have to focus my hours finding revenue‐generating sources to keep my nonprofit afloat.

Participants also discussed how administrative processes within the university system remain a challenge, as traditional institutional practices often hinder effective community engagement. An academic partner said:

I think this [the STOP COVID‐19 CA network] shows that maybe there is finally a way for universities to recognize that the current ‘business as usual’ approach is not working. It is damaging relationships, especially when we position ourselves as community‐based scholars. This damage affects both the reputation of the institution and our work with communities.

Structural barriers within universities limited the implementation of equitable research practices. Another academic partner pointed out:

Even if all the intentions are there, which is not always the case, structural barriers still exist that make it hard to conduct research the way we want to. The payment structure for CBOs and participants is one major obstacle.

These barriers further undermine the impact of community–academic partnerships as they limit resource sharing among partners. A community partner emphasised how the bureaucracy of large university educational systems hampers the impact of community–academic partnerships:

I would like to highlight the institutional barriers to partnership, especially around resource‐sharing and compensation for community organizations and research participants. Many of us are working within our institutions to address these challenges. Since many of us are part of the UC system, solutions might come from the UC Office of the President, who could help remove these barriers or prioritize the removal of obstacles to partnership and compensation at a regional or system‐wide level.

Participants also talked about the need for structural changes within local and regional public health departments to ensure culturally responsive public health practices. Despite lessons learned about the importance of linguistically and culturally relevant strategies as part of the alliance, the response of public health in the face of a new infectious disease near the end of the pandemic (i.e., monkeypox) demonstrated a lack of adaptability and inclusion of evidence‐based findings generated via the alliance. An academic partner said:

Monkeypox came so quickly after COVID, and despite all the recommendations and lessons learned that we and other community members shared, the city and county went back to almost the exact same approach—English‐only mass testing and vaccination sites. This was against all the best practices we collectively developed in partnering with communities.

This perspective highlighted a gap in translation of evidence to practice, whereby previous knowledge regarding the inclusion of non‐English speaking communities in public health education and promotion was not put into practice, undermining efforts to engage diverse communities in public health mitigation efforts. Participants stressed the need for counties to adopt inclusive, multilingual strategies that reflect lessons learned from prior public health crises, such as the Covid‐19 pandemic.

## 4 Discussion and Implications

The use of REM in the evaluation of the STOP COVID‐19 CA project/NIH CEAL initiative provided a nuanced understanding of the impact of the project on short‐, medium‐ and long‐term outcomes. Our findings indicate that most outcomes were observed at the individual and interpersonal (group) levels and involved primarily medium‐term changes, such as modifications in partner behaviours and group dynamics. This suggests that the STOP COVID‐19 CA network was most effective at facilitating behavioural shifts among individuals and within small groups, rather than producing structural‐ or policy‐level transformations. These findings suggest that further structural support or policy interventions might be needed to extend the impact of community–academic partnerships, such as those within the network established during the Covid‐19 pandemic, to result in long‐term impacts.

The findings suggest that participatory methods like REM are ideal for evaluating complex, multi‐stakeholder initiatives as they reveal the interconnectedness of individual, community and institutional changes [25]. The SEM contextualised the findings across the multiple levels of influence shaping human behaviour and changes over time [25, 26, 32]. Engagement across multiple spheres of influence was particularly important for community‐engaged research addressing Covid‐19‐related health disparities, as it fostered opportunities for interventions tailored to the specific needs and resources of local populations promoting both individual and collective well‐being. An example is the development of restorative circles, a community mental health intervention led by a community–academic partnership employing anti‐racist praxis to address traumas linked to the Covid‐19 pandemic [33].

By integrating insights from diverse collaborators, including CHWs, community leaders and academics, our analysis captured both intended and unintended changes as part of community–academic partnerships. Consistent with the findings of Zimmerman et al. [24], the REM method facilitated an iterative process of stakeholder reflection and storytelling, highlighting the impact of Covid‐19 interventions on community behaviours and perceptions about public health initiatives in California. For instance, the STOP COVID‐19 CA network increased awareness of the CHW/promotor(a) model and its value in building trust in public health mitigation strategies (testing and vaccination) and addressing health inequities in historically underserved communities [21].

Our findings align with findings from Bloom [34], who demonstrated that REM effectively highlights how community development initiatives enhance local capacity, fostering sustainable change and identification of the wider impacts of public health interventions [35]. For instance, at the level of policy and built environment, research carried out through the various projects in the STOP COVID‐19 CA network attempted to flatten hierarchies within academic institutions, pushing institutions to reconsider their approaches to compensating the community and resource‐sharing and to move towards more equitable practices. Participants' reflections reveal the need for universities to reevaluate their systems and practices to support genuine community partnerships and equitable compensation. As noted by Burke et al. [36], fostering decolonised fiscal relationships between universities and community organisations is essential for the success of such collaborations.

The use of REM to identify inequitable fiscal and resource‐sharing practices and contextualisation of these practices within the policy and built environment of the SEM provided a comprehensive understanding of systems‐level transformations and the administrative and infrastructure needs of community–academic partnerships. Institutions of higher education shape the CBPR process by creating obstacles (albeit often unintentionally) to engaging the community in the academy via research and calling attention to the need for more equitable structures in academia and academic medical centres. Research carried out during the Covid‐19 pandemic via the statewide network shed light on the need to decolonise fiscal relationships between universities and community organisations [36]. By decolonising fiscal relationships, universities can move towards more equitable partnerships and ensure the voices of marginalised communities are central in the research and policy‐making processes.

The REM method offers significant strengths in both general applications and specific projects like those carried out throughout the STOP COVID‐19 CA network. One of the primary strengths of REM lies in its participatory nature, allowing diverse voices and perspectives to collectively identify and articulate the diverse impacts of interventions that took place in their communities. This inclusive and participatory approach not only enhances the validity of findings but also fosters a sense of ownership and empowerment among participants, ultimately leading to more sustainable outcomes and long‐term partnerships. As Washburn et al. [37] noted, REM facilitates the identification of diverse perspectives among programme implementers, who, in our study, included CHWs, CBOs, healthcare and public health partners, and academics, enriching the evaluation process. Such a multi‐perspective and comprehensive evaluation reinforces the importance of using participatory methods like REM to evaluate cross‐sector collaborations and inform future public health initiatives and policies [38].

While this method deepened understanding of the relationships between the STOP COVID‐19 CA network and short‐term changes in knowledge, skills and attitudes; medium‐term changes in awareness, capacity and trust among historically marginalised communities; and the long‐term conditions for change to occur, the findings should be interpreted considering several limitations. REM requires considerable time and resources to facilitate group discussions and synthesise data and insights. The ideal size of an REM session is 8–12 participants—a size that permits sufficient time for group activities (e.g., sharing out and reflecting) during sessions, which are typically completed within 2 h [39]. We structured our sessions to include a 10‐min introduction followed by 10 min of appreciative inquiry, ~75 min of sharing out, and 10 min of reflection, interspersing pockets of 2–3 min for transition between activities (e.g., movement from breakout rooms to main session). We would recommend providing more time for the appreciative inquiry peer interviews, as this activity asks participants to talk about themselves and their experiences, setting the tone for subsequent group activities [39]. All participants approved the map of the session they attended. After the individual maps were accepted by participants, we created one final map with findings from the three sessions.

Furthermore, the mapping process can be challenging as facilitators or ‘mappers’ need to use mind‐mapping techniques to organise key points shared in real time. On a related theme, the initial maps did not strictly adhere to REM guidelines, particularly in the application of theory and three types of changes (individual‐, medium‐ and long‐term) as outlined by the Web Mapping variation of the REM approach [35]. To ensure alignment with both the REM guidelines (categorising changes by type) and the analytical framework of the SEM model, we subsequently reorganised the maps to accurately reflect the discussions and analytical structure and shared the maps with the research participants to include their recommendations and feedback. If choosing to follow the Web Mapping variation of REM, we recommend identifying the framework and organising the sessions to focus on change across the three distinct levels.

Another limitation of our data is the over‐representation of community and academic partners from a single site in the Spanish‐language session. While the network included several community organisations that served Spanish‐speaking communities, there were only a few in leadership positions within these organisations that spoke Spanish and were interested in participating in the research. Similarly, the pool of Spanish‐speaking academic partners was small, limiting recruitment to less than a third of the academic sites. Last, we collected data from participants in one REM session only. As others have noted, a one‐time approach to collecting data at the end of project implementation may limit findings to an overly positive and simplistic understanding of change and impact. As Nobles et al. [35] point out, longitudinal data collection involving multiple REM sessions can identify changes as well as anticipated changes within systems and their actual manifestation over time. Despite these limitations, the use of REM to assess the impact of participation in the statewide network on the capacity of community–academic partnerships to carry out Covid‐19‐related health disparities research was valuable. This method captured the nuanced experiences and perspectives of diverse members of the network (e.g., Spanish‐speaking community collaborators, research staff, student research assistants and principal investigators), thereby fostering a deeper understanding of the impact of the network on partnerships' capacity to address health disparities via research during the pandemic.

## 5 Conclusion

Involvement in the STOP COVID‐19 CA network had wide‐ranging and multilevel impacts on the capacity of community–academic partnerships to carry out Covid‐19‐related health disparities research. The findings underscore the importance of cross‐sector collaborations in fostering trust, promoting equitable health practices and advancing the public health impact of community–academic partnerships. The project not only addressed immediate health needs related to Covid‐19, such as access to Covid‐19 testing sites and vaccines, but also put into motion long‐term structural changes needed to support ongoing community engagement in health equity research. This network serves as a model for community–academic partnerships focused on addressing health disparities in socio‐culturally, linguistically and geographically diverse communities [9].

## Author Contributions

**Evelyn Vázquez:** conceptualization, data curation, formal analysis, investigation, methodology, validation, visualization, writing – original dtaft, review and editing. **Isabel Duong:** data curation, formal analysis, investigation, visualization, writing – original draft, review and editing. **Borsika A. Rabin:** conceptualization, formal analysis, investigation, writing – original draft, review and editing. **Nicole A. Stadnick:** conceptualization, formal analysis, funding acquisition, investigation, writing – original draft, review and editing. **Paul L. Watson Jr.:** conceptualisation, funding acquisition, investigation, methodology, writing – original draft, review and editing. **Kelli L. Cain:** investigation, project administration, writing – original draft, review and editing. **Maria Pozar:** conceptualization, funding acquisition, investigation, validation, writing – original draft, review and editing. **Ann Cheney:** conceptualisation, data curation, formal analysis, funding acquistion, investigation, methodology, project administration, supervision, writing – original draft, review and editing.

## Ethics Statement

All research activity was approved by the University of California Riverside Institutional Review Board via human subjects protocol # 30036.

## Conflicts of Interest

The authors declare no conflicts of interest.

## Data Availability

De‐identified data are available via request from the corresponding author.
